# Supplementary material for: Advanced imaging and trends in hospitalizations from the emergency department
Source: PLoS One. 2020 Sep 16;15(9):e0239059. doi: 10.1371/journal.pone.0239059 (PMC7494122; doi:10.1371/journal.pone.0239059)
Supplement: S1 Table — (DOCX) [file pone.0239059.s001.docx]

| **S1 Table. Definition of presenting complaints by NCHS reason for visit codes** | |
| --- | --- |
| **Presenting Complaint** | **Reason for Visit Code** |
| Injury | 5000.x–5830.x, 5838.x |
| Psychiatric | 1100.x–1199.x |
| Upper Respiratory | 1345.x–1365.x, 1400.x–1410.x, 1425.x, 1435.x–1480.x |
| Abdominal Pain | 1545.x |
| Leg Symptoms | 1915.x–1935.x |
| Chest pain | 1050.x |
| Neck/Back pain | 1900.x–1910.x |
| Fever | 1005.x, 1010.x |
| Nausea/Vomiting/Diarrhea | 1525.x, 1530.x, 1595.x |
| Shortness of Breath | 1415.x, 1420.x, 1430.x |
| Arm Symptoms | 1940.x–1960.x |
| Headache | 1210.x, 2365.x |
| Skin Complaints | 1830.x–1899.x |
| Dizziness/syncope | 1030.x, 1225.x |
| Pregnancy Problems | 1790.x, 1791.x |
| Flank Pain | 1055.2 |
| General Weakness | 1015.x, 1020.x |
| Neurological Symptom | 1095.x, 1220.x, 1230.x, 1235.x, 1305.x |
| Convulsions | 1200.x, 1205.x |
| Vaginal Bleeding | 1730.x–1755.x |
| Abbreviations: NCHS, National Center for Health Statistics | |
