# Supplementary material for: Advanced imaging and trends in hospitalizations from the emergency department
Source: PLoS One. 2020 Sep 16;15(9):e0239059. doi: 10.1371/journal.pone.0239059 (PMC7494122; doi:10.1371/journal.pone.0239059)
Supplement: S2 Table — (DOCX) [file pone.0239059.s002.docx]

| **S2 Table. Changes in adjusted admission rates comparing 2007-2008 to 2015-2016, only inpatient hospitalization considered admission.** | | | | | | | | | | | | | | | | | | | |
| --- | --- | --- | --- | --- | --- | --- | --- | --- | --- | --- | --- | --- | --- | --- | --- | --- | --- | --- | --- |
|  | Visits with Imaging | | | | Visits without Imaging | | | | | | | | | | | |  |  |  |
| **Presenting complaint** | Adjusted Admission Rates (%) | | Adjusted RR (95% CI) | | | Adjusted Admission Rates (%) | | | | | | Adjusted RR (95% CI) | | | | | Relative Difference,  % (95% CI) | | |
|  | 2007-8 | 2015-16 |  |  | | 2007-8 | | 2015-16 | | |  | | |  | |  | |  | p |
| Overall | 19.8 | 14.9 | 0.75 | (0.67, 0.84) | | | 12.3 | | 9.9 | 0.8 | | | (0.72, 0.88) | | -5.1 | | | (-13.2, 3.0) | 0.21 |
| Injury | 11.0 | 7.8 | 0.71 | (0.52, 0.90) | | | 5.2 | | 4.8 | 0.94 | | | (0.73, 1.15) | | -23.1 | | | (-48.7, 2.5) | 0.08 |
| Psychiatric | 21.8 | 18.7 | 0.86 | (0.67, 1.05) | | | 17.8 | | 18.8 | 1.05 | | | (0.87, 1.24) | | -19.6 | | | (-43.9, 4.8) | 0.12 |
| Upper Respiratory | 16.6 | 11.0 | 0.67 | (27.9, 105.1) | | | 4.3 | | 3.1 | 0.74 | | | (0.52, 0.96) | | -7.1 | | | (-47.1, 32.8) | 0.73 |
| Abdominal Pain | 25.3 | 16.0 | 0.63 | (0.52, 0.75) | | | 12.8 | | 7.2 | 0.56 | | | (0.41, 0.72) | | 6.9 | | | (-11.8, 25.6) | 0.47 |
| Leg Symptoms | 16.5 | 9.9 | 0.60 | (0.35, 0.86) | | | 8.6 | | 6.5 | 0.76 | | | (0.54, 0.98) | | -15.8 | | | (-50.1, 18.5) | 0.37 |
| Chest pain | 36.3 | 24.2 | 0.67 | (0.51, 0.82) | | | 29.0 | | 19.3 | 0.67 | | | (0.54, 0.79) | | 0.0 | | | (-16.2, 16.2) | 0.99 |
| Neck/Back pain | 9.2 | 8.2 | 0.89 | (0.44, 1.35) | | | 4.0 | | 2.0 | 0.49 | | | (25.9, 73.2) | | 40.4 | | | (-7.3, 88.1) | 0.10 |
| Fever | 22.4 | 16.7 | 0.74 | (0.33, 1.15) | | | 7.6 | | 7.3 | 0.96 | | | (0.72, 1.20) | | -21.9 | | | (-68.5, 24.8) | 0.36 |
| Nausea/Vomiting/Diarrhea | 21.7 | 17.2 | 0.79 | (0.57, 1.02) | | | 14.2 | | 9.1 | 0.64 | | | (0.45, 0.83) | | 15.6 | | | (-11.2, 42.3) | 0.26 |
| Shortness of Breath | 41.5 | 43.9 | 1.06 | (0.80, 1.32) | | | 36.1 | | 30.6 | 0.85 | | | (0.73, 0.96) | | 21.2 | | | (-3.2, 45.6) | 0.09 |
| Arm Symptoms | - | - | - | - | | | 3.3 | | 3.2 | 0.96 | | | (0.58, 1.34) | | - | | | - | - |
| Headache | 7.4 | 7.8 | 1.05 | (0.48, 1.61) | | | 3.0 | | 4.4 | 1.45 | | | (0.52, 2.37) | | -39.7 | | | (-140.5, 61.1) | 0.44 |
| Skin Complaints | - | - | - | - | | | - | | - | - | | | - | | - | | | - | - |
| Dizziness, syncope | 27.0 | 19.5 | 0.72 | (0.51, 0.93) | | | 19.8 | | 9.8 | 0.49 | | | (0.34, 0.65) | | 22.7 | | | (-2.9, 48.3) | 0.08 |
| Pregnancy Problems | - | - | - | - | | | 15.0 | | 14.0 | 0.94 | | | (0.42, 1.45) | | - | | | - | - |
| Flank Pain | 10.6 | 7.8 | 0.74 | (0.38, 1.11) | | | 10.0 | | 4.3 | 0.43 | | | (0.14, 0.73) | | 30.8 | | | (-15.6, 77.3) | 0.19 |
| General Weakness | 48.9 | 46.2 | 0.95 | (0.72, 1.17) | | | 38.2 | | 27.8 | 0.73 | | | (0.56, 0.90) | | 21.6 | | | (-5.9, 49.0) | 0.12 |
| Neurological Symptom | 38.6 | 29.1 | 0.75 | (0.54, 0.96) | | | 13.0 | | 14.8 | 1.14 | | | (0.55, 1.73) | | -38.6 | | | (-100.0, 22.9) | 0.22 |
| Convulsions | 28.2 | 16.1 | 0.57 | (0.30, 0.84) | | | 16.2 | | 6.4 | 0.39 | | | (0.16, 0.62) | | 17.7 | | | (-17.1, 52.6) | 0.32 |
| Vaginal Bleeding | - | - | - | - | | | - | | - | - | | | - | | - | | | - | - |
| Note: Adjusted admission rates, adjusted risk ratios, and absolute differences calculated from survey-weighted multivariable logistic regression and marginal estimating methods. All models adjusted for patient and visit characteristics. A negative absolute difference means admission rate reduced by greater proportion among imaged visits compared to visits without advanced imaging. Omitted admission rates (and the corresponding adjusted RR and absolute difference, had <30 observations, which were considered unreliable as recommended by the National Center for Health Statistics.  Abbreviations: RR, risk ratio; CI, confidence interval. | | | | | | | | | | | | | | | | | | | |
