# Supplementary material for: Advanced imaging and trends in hospitalizations from the emergency department
Source: PLoS One. 2020 Sep 16;15(9):e0239059. doi: 10.1371/journal.pone.0239059 (PMC7494122; doi:10.1371/journal.pone.0239059)
Supplement: Attachment — Submitted filename: Reviewer comments and response.docx [file pone.0239059.s004.docx]

Reviewer #1: This manuscript is very well-written and addresses an important topic regarding healthcare resource utilization. Having said that, this issue is complex and I have a few suggestions that would potentially help the paper reflect this complexity.

1. Hypothesis
I think your hypothesis - that advanced ED imaging would lead to a decline in admission rates - needs a bit more rationale earlier in the paper. Intuitively, and as you later mention in the discussion, advanced imaging could instead lead to an increase in hospitalization if more subtle but possibly dangerous pathology was identified. Although your paper did not show any statistical difference for imaging vs. hospitalization in either direction, I think some justification for your hypothesis would help the reader follow the paper.

Thank you for this suggestion, we reorganized the introduction to refocus the study’s emphasis on examining the value of increased advanced imaging through reduction in hospital admissions.

2. Confounding variables
There are several variables that affect the decision to admit a patient to hospital. Of course, the disposition of patients at either end of the spectrum are obvious (ie. a fractured toe vs. a patient in septic shock), but much of emergency medicine operates in a grey area where two different providers could make two different, and equally justifiable, decisions. This is difficult to capture in a binary "admitted vs. discharge" outcome measure.

Additionally, admission can sometimes be a product of patient preference or lack of outpatient supports. I think that you have done a good job explaining some of these variables in the conclusion ("outpatient clinical pathways, and policy factors...") but can better underscore the many factors which are at play in the decision to admit a patient to hospital, and include this is a major limitation to your study.

In this revision, we acknowledge the limitation of our data to account for the complex decision of admitting a patient more explicitly.

*“Furthermore, we acknowledge that the decision of whether hospitalize after an ED evaluation is complex may not be fully accounted for in our analysis.”*

3. Methods
How were patients who were seen in the ED and then discharged with a plan/appointment to return the next day for advanced imaging dealt with? Was this information reflected in the database? This is a relatively common practice in the EDs I have worked at for patients seen in the evening/overnight without critically emergent differential diagnoses.

We are not aware that planned return ED visits is a commonplace practice, at least in the community sites where we work. Nevertheless, the NHAMCS data is consisted of a cross-sectional sample of ED visits from EDs that were surveyed annually. The patients are thus not followed longitudinally across visits. Therefore, NHAMCS is unable to identify visits that may be “return” or “repeat” visits. We additionally noted this limitation as considerations for imaging use would certainly be different for repeat or return visits.

*“The cross-sectional nature of the dataset also does not allow us to discern whether visits may be return or repeat ED visits where decisions to pursue advanced imaging would be different from initial ED visits.”*

4. Results
No further suggestions here - I was happy to see you include relevant high-level results for subgroups (ie. excluding observation admissions and excluding ultrasound) - I think this adds credence to the arguments brought forth in the paper.

Thank you.

5. Conclusion
You suggest that advanced imaging may not play a substantial role in the decline of admission rates. Despite your well-executed study, I am still not convinced of this, largely because of the many confounding variables, and so I think it would be more appropriate to state that more research is needed.

We adjusted our tone in the CONCLUSION section to be more align our finding as preliminary and needs further replication. However, we do believe that our study design is stronger exploratory compared to studies that simply observed that the decline in admission rates and the increase in advanced imaging occurred concurrently.
